# Leveraging transcription factor physical proximity for enhancing gene regulation inference

**DOI:** 10.1093/bioinformatics/btaf186

**Published:** 2025-07-15

**Authors:** Xiaoqing Huang, Aamir R Hullur, Elham Jafari, Kaushik Shridhar, Mu Zhou, Kenneth Mackie, Kun Huang, Yijie Wang

**Affiliations:** Department of Biostatistics and Health Data Sciences, Indiana University School of Medicine, Indianapolis, IN 46202, United States; Computer Science Department, Indiana University Bloomington, Bloomington, IN 47408, United States; Computer Science Department, Indiana University Bloomington, Bloomington, IN 47408, United States; Computer Science Department, Indiana University Bloomington, Bloomington, IN 47408, United States; Department of Computer Science, Rutgers University, New Brunswick, NJ 08910, United States; Gill Center for Biomolecular Science, Department of Psychological and Brain Sciences, Indiana University Bloomington, Bloomington, IN 47408, United States; Department of Biostatistics and Health Data Sciences, Indiana University School of Medicine, Indianapolis, IN 46202, United States; Computer Science Department, Indiana University Bloomington, Bloomington, IN 47408, United States

## Abstract

**Motivation:**

Gene regulation inference, a key challenge in systems biology, is crucial for understanding cell function, as it governs processes such as differentiation, cell state maintenance, signal transduction, and stress response. Leading methods utilize gene expression, chromatin accessibility, transcription factor (TF) DNA binding motifs, and prior knowledge. However, they overlook the fact that TFs must be in physical proximity to facilitate transcriptional gene regulation.

**Results:**

To fill the gap, we develop GRIP—Gene Regulation Inference by considering TF Proximity—a gene regulation inference method that directly considers the physical proximity between regulating TFs. Specifically, we use the distance in a protein–protein interaction (PPI) network to estimate the physical proximity between TFs. We design a novel Boolean convex program, which can identify TFs that not only can explain the gene expression of target genes (TGs) but also stay close in the PPI network. We propose an efficient algorithm to solve the Boolean relaxation of the proposed model with a theoretical tightness guarantee. We compare our GRIP with state-of-the-art methods (SCENIC+, DirectNet, Pando, and CellOracle) on inferring cell-type-specific (CD4, CD8, and CD 14) gene regulation using the PBMC 3k scMultiome-seq data and demonstrate its out-performance in terms of the predictive power of the inferred TFs, the physical distance between the inferred TFs, and the agreement between the inferred gene regulation and PCHiC data.

**Availability and implementation:**

https://github.com/EJIUB/GRIP.

## 1 Introduction

The transcriptional regulatory program in cells is essential for maintaining their specific functions, fitness, and responses to environmental changes. This program involves complex interactions between transcription factors (TFs), enhancers, and target genes (TGs). Many state-of-the-art methods have been developed to uncover such cell-type-specific transcriptional regulation programs by solely using gene expression data. However, DREAM5 network inference challenge (Marbach *et al.* 2012) and a recent benchmarking result for single-cell gene regulation inference (BEELINE) (Pratapa *et al.* 2020) demonstrated that methods that only use gene expression data are far from solving the gene regulation inference problem, suggesting that relying merely on gene expression is not enough. To improve the inference power, prior-based methods (Lam *et al.* 2016, Duren *et al.* 2017, Siahpirani and Roy 2017, Wang *et al.* 2018, Miraldi *et al.* 2019, Duren *et al.* 2020, Bachireddy *et al.* 2021, Duren *et al.* 2021, Xu *et al.* 2021, Fleck *et al.* 2022, Gibbs *et al.* 2022, Jiang *et al.* 2022, Kartha *et al.* 2022, Zhang *et al.* 2022, Bravo González-Blas *et al.* 2023, Kamal *et al.* 2023, Kamimoto *et al.* 2023, Li *et al.* 2023, Zhang *et al.* 2023) have been proposed to utilize existing additional context-specific multi-omics data and prior knowledge. These methods incorporate gene expression data either with epigenomics data (such as chromatin accessibility data and chromatin immunoprecipitation data), and/or with gene knockout data, and/or with known TF–TG regulation from previous studies.

However, there are no existing methods that directly consider the fact that TFs need to be in physical proximity to accomplish transcriptional regulation. From previous studies (Ma 2011, Lambert *et al.* 2018, Mattaini 2020, Richter *et al.* 2022), we know that in eukaryotic cells, TFs must work together, often forming complexes or interacting with co-factors/mediators, to effectively regulate gene expression. Overlooking this aspect of TF cooperation might lead to the identification of putative TFs that are impossible to be in physical proximity through either direct interactions or interactions with the co-factors/mediators. Methods that leverage the physical proximity between regulating TFs would have the advantage of incorporating this constraint when inferring gene regulation, potentially uncovering the correct regulatory TFs even in cases where gene expression data is noisy. Considering TFs physical proximity could improve the accuracy of gene regulation inference and offer more biologically plausible insights into the mechanisms of transcriptional regulation.

To fill the gap, in this article, we develop Gene Regulation Inference by considering TF Proxmity (GRIP), a mathematically rigorous method, which builds on a novel sparse learning model with Boolean variables that considers the physical proximity between TFs. Specifically, GRIP is designed to model the transcriptional regulation for a TG using paired gene expression and chromatin accessibility data. The paired data provide the distal open regions around the TG, the potential TFs that could bind to those distal open regions, and the expression of the TG and potential TFs. GRIP ought to find the best TFs whose expression can explain the expression of TG and are in physical proximity, which can be estimated by the distance in a protein–protein interaction (PPI) network. By considering both the regulatory relationship and the spatial constraints, GRIP has the potential to enhance the accuracy and relevance of gene regulation inference. Mathematically, GRIP is formulated as a Boolean convex program. We propose to use the projected quasi-Newton method to efficiently solve the Boolean relaxation of GRIP. In addition, we provide the necessary and sufficient conditions for Boolean relaxation to achieve the exactness of the original Boolean convex program.

To evaluate the performance of GRIP, we compare it with the state-of-the-art methods, including Scenic+ (Bravo González-Blas *et al.* 2023), Pando (Fleck *et al.* 2022), CellOracle (Kamimoto *et al.* 2023), and DirectNet (Zhang *et al.* 2022). We apply all the competing methods to infer cell-type-specific gene regulation using scMultiome-seq data of CD4 T cells, CD8 T cells, and CD14 monocytes, which are extracted from scMultiome-seq PBMC 3k data (10x Genomics 2021). After benchmarking the competing methods, we find that GRIP outperforms the others, as the TFs inferred by GRIP demonstrate greater predictive power in forecasting TG gene expression. Furthermore, we observe that the TFs inferred by GRIP remain close in the PPI networks compared to those identified by the competing methods. Last but not least, we use PCHiC data (Javierre *et al.* 2016) in naive CD4 cells, naive CD8 cells, and monocytes to evaluate all the competing methods. We find that the results generated by GRIP are in better agreement with the PCHiC data.

Our GRIP model fills an important gap in gene regulation inference by incorporating the critical aspect of physical proximity between TFs. As a spin-off, the Boolean convex program and its relaxation have the potential to be applied to other computational biology problems.

## 2. Materials and methods

Before describing the mathematical foundations behind the GRIP model, we first review the transcriptional regulation in eukaryote cells to introduce the biological foundation of the GRIP model, which is the hypothesis that TFs regulating the same TGs are close to each other in the PPI network than randomly selected TFs. We then use publicly available data to empirically demonstrate the hypothesis holds. Next, we introduce how we incorporate the hypothesis into a sparse learning model to formulate the GRIP model as a Boolean convex program. We applied Boolean relaxation to the Boolean program and proved that our Boolean relaxation was tight (a Theorem provides the necessary and sufficient conditions for the relaxed problem to yield the solution of the original problem). In the end, we propose to use the projected quasi-Newton method to efficiently solve the relaxed model and a rounding technique to retrieve the Boolean solutions.

### 2.1 TF proximity in transcriptional gene regulation

**Figure 1.**
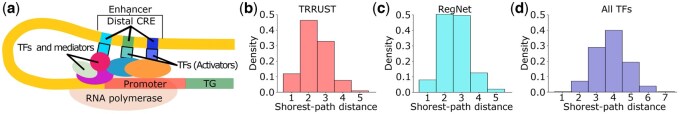
TF proximity in transcriptional gene regulation. (a) Illustration of how TFs (including TFs, mediators, and activators) work together in transcriptional gene regulation. CRE stands for cis-regulatory element. (b) The empirical distribution of the shortest-path distance between TFs of TGs in TRRUST (Han *et al.* 2018). (c) The empirical distribution of the shortest-path distance between TFs of TGs in RegNetwork (Liu *et al.* 2015). (d) The empirical distribution of the shortest-path distance between all pairs of TFs in humans extracted from (Lambert *et al.* 2018).

In general, eukaryotic TFs must work together to achieve needed specificity in both DNA binding and effector function (Ma 2011, Lambert *et al.* 2018, Richter *et al.* 2022). As shown in Fig. 1a, the DNA folds such that the enhancer is brought into proximity with the promoter, allowing interaction between the activators (a special type of TF) and the TFs that form the transcription initiation complex (Mattaini 2020). Therefore, we hypothesize that TFs regulating the same TGs tend to be physically closer than random TFs. We will empirically test our hypothesis in the following section.

### 2.2 Hypothesis test using PPI proximity

We test the hypothesis that TFs regulating the same TGs tend to be physically closer than random TFs through network analysis. We first extract known TFs of TGs in humans from TRRUST (Han *et al.* 2018) and RegNetwork (Liu *et al.* 2015). We further extract physical PPIs from BioGRID (Oughtred *et al.* 2019) and construct a PPI network. We use the shortest-path distance in a PPI network to measure the physical closeness between TFs. In Fig. 1b and c, we show the empirical distributions of the shortest-path distance between known TFs of the same TGs in TRRUST (Han *et al.* 2018) and RegNetwork (Liu *et al.* 2015), respectively. Furthermore, in Fig. 1d, we show the empirical distribution of the shortest-path distance between all pairs of TFs in humans extracted from Lambert *et al.* (2018). We compute the shortest-path distance between all pairs of TFs and plot the histogram. This distribution can be considered as the background distribution.

Comparing the distribution in Fig. 1b and the distribution in Fig. 1d, it is clear that the average shortest-path distance between known TFs of TGs in TRRUST (Han *et al.* 2018) is significantly smaller than the average shortest-path distance between all pairs TFs in humans (*P*-value <1e−10, Mann–Whitney *U* test with tie correction). Similarly, after comparing the distribution in Fig. 1c and the distribution in Fig. 1d, we find that the average shortest-path distance between known TFs of TGs in RegNetwork (Liu *et al.* 2015) is significantly smaller than the average shortest-path distance between all pairs TFs in humans (*P*-value <1e−6, Mann–Whitney *U* test with tie correction).

In sum, we use the TFs’ distance in the PPI network to demonstrate the hypothesis that TFs regulating the same TGs are in physical proximity holds. In the next section, we will show how we incorporate the hypothesis into our GRIP model.

### 2.3 The formulation of the GRIP model

#### 2.3.1 Sparse learning model

**Figure 2.**
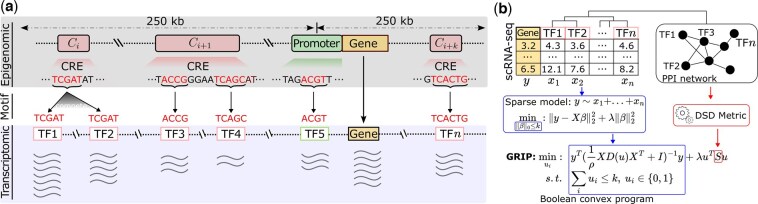
The overview of our GRIP. (a) Paired measurements on the epigenomic layer and transcriptomic layer and their relationship. Motif data are the bridge. CRE stands for cis-regulatory elements. Our GRIP model takes such paired measurements as input. (b) The GRIP model integrates the sparse model, which selects the TFs whose expression can explain TG expression, and $u^{T}Su$, which makes sure the selected TFs stay close in the PPI network.

We first assume that we have the paired gene expression and chromatin accessibility data (as shown in Fig. 2a). One example of such data is scMultiome, where scRNA-seq and scATAC-seq are measured simultaneously. We can use chromatin accessibility data and TF motif data to find TF candidates that could bind to the open chromatin regions around a TG, as illustrated in Fig. 2a, using FIMO in the MEME suite (Bailey *et al.* 2015). We can obtain these TFs’s gene expression $x_{1},\ldots ,x_{n}$ (where $x_{i}\in R^{m}$ and *m* is the sample size/number of cells) and the TG’s gene expression $y\in R^{m}$ (as shown in Fig. 2b). We can then model the gene regulation of the TG using a linear model: $y=\beta_{1}x_{1}+\cdots +\beta_{n}x_{n}+\epsilon$. However, as illustrated in Fig. 2a open region $C_{i}$, more than one TF could compete for one genome location. In addition, one open region could have several TFs bound (as shown in Fig. 2a, open region $C_{i+1}$). According to the “futility theorem”, unbound instances of a TF’s cognate motif will typically greatly outnumber those that are occupied by the TF (Lambert *et al.* 2018), implying motif matches do not mean true binding. To identify the underlying TFs for the TG, we propose to use the sparse learning model (1) to find the *k* most possible TFs (as shown in Fig. 2b).


(1) $$\underset{∥\beta {∥}_{0}\leq k}{\min}:∥y-X\beta {∥}_{2}^{2}+\rho ∥\beta {∥}_{2}^{2}$$

In the model, $\beta ={\left[\beta_{1},\ldots ,\beta_{n}\right]}^{T}$ is the coefficient of the linear model, $X=[x_{1},\ldots ,x_{n}]\in R^{m\times n}$ is the design matrix containing gene expression of TF candidates, and *y* is the response vector containing the expression of the TG. The constraint $∥\beta {∥}_{0}\leq k$ ($∥\cdot {∥}_{0}$ is the $\ell_{0}$ norm that counts the number of non-zero elements) enforces to only pick *k* important TFs whose expression can better explain the gene expression of the TG *y*. And these *k* important TFs are interpreted as the potential TFs that regulate the TG.

We notice that the sparse model (1) is a non-convex optimization problem due to using the $\ell_{0}$ norm in the constraint $∥\beta {∥}_{0}\leq k$. From previous work (Pilanci *et al.* 2015, Ang *et al.* 2021), we know the non-convex optimization problem (1) is mathematically equivalent to the Boolean program written in (2)


(2) $$\begin{array}{c} \underset{u_{i}}{\min}:\;\;y^{T}{\left(\frac{1}{\rho }XD(u)X^{T}+I\right)}^{-1}y\\ \;\;\;\;\;\;s.t.\;\;\sum_{i}u_{i}\leq k,\;u_{i}\in \{0,1\} \end{array}$$

where $u_{i}$ is a Boolean variable that needs to be optimized and D(u) is a diagonal matrix with $u={\left[u_{1},\ldots ,u_{n}\right]}^{T}$ on its diagonal. Importantly, $u_{i}$ in (2) is closely related to $\beta_{i}$ in (1). Mathematically, $u_{i}=1$ means $\beta_{i}\neq 0$, which denotes that the gene expression $x_{i}$ of TF *i* is selected by the sparse model; $u_{i}=0$ means $\beta_{i}=0$, which indicates that the gene expression $x_{i}$ of TF *i* is not selected by the sparse model. Therefore, $u_{i}\in \{0,1\}$ is the indication of whether TF *i* is selected by the sparse model (1). In the following section, we take advantage of the indication property of $u_{i}\in \{0,1\}$ to incorporate the distance between TFs in a PPI network into our sparse model (2).

#### 2.3.2 Incorporate TF proximity

To make sure the TFs selected by the sparse model (2) are also close to each other in a PPI network, we propose our GRIP model (3) as follows:


(3) $$\begin{array}{c} P:\;\;\underset{u_{i}}{\min}:y^{T}{\left(\frac{1}{\rho }XD(u)X^{T}+I\right)}^{-1}y+\lambda u^{T}Su\\ \;\;\;\;\;\;\;\;\;\;\;s.t.\sum_{i}u_{i}\leq k,\;u_{i}\in \{0,1\} \end{array}$$

Compared with the sparse model (2), the GRIP model (3) adds a term $u^{T}Su$ to (2) and use λ to balance them (as shown in Fig. 2b). The term $u^{T}Su$ computes the distance between selected TFs in a PPI network. Through combining the sparse model (2) and the term $u^{T}Su$, the GRIP model can identify TFs that can not only explain the gene expression *y* of the TG but also stay close to each other in a PPI network.

**Figure 3.**
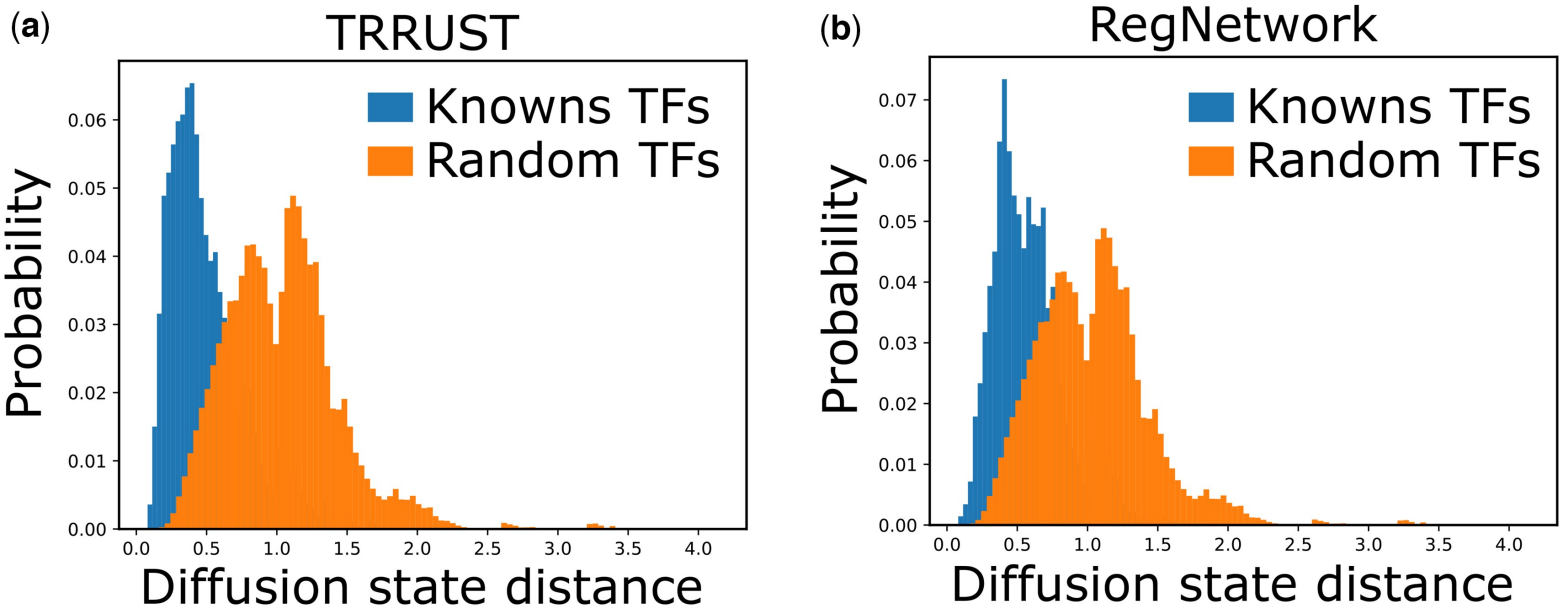
Hypothesis test using DSD metric. (a) Using known TFs in TRRUST (Han *et al.* 2018). (b) Using known TFs in RegNetwork (Liu *et al.* 2015).

In term $u^{T}Su$ of (2), *S* is a distance matrix, where $S_{ij}$ is the distance between TF *i* and TF *j* in the PPI network. Remarkably, we use the diffusion state distance (DSD) metric (Cao *et al.* 2013) (as shown in Fig. 2b) rather than the shortest-path distance metric to compute the distance between TFs, because the DSD metric is symmetric, positive definite (matrix *S*), and obeys the triangle inequality. We find that our hypothesis in Section 2.2 still holds if we use the DSD metric (as shown in Fig. 3). For both (a) and (b) in Fig. 3, clearly, the average DSD distance between known TFs of TGs is significantly smaller than the average DSD distance of all pairwise TFs (*P*-value <1e−3, Mann–Whitney *U* test with tie correction).

Notably, the GRIP model (P) (3) is a Boolean convex program, where the objective function is convex and the variables $u_{i}\in \{0,1\},\;\forall i\in \{1,2,\ldots ,n\}$ that need to be optimized are Boolean variables.

### 2.4 Boolean relaxation of the GRIP model and its exactness

Due to the combinatorial nature of the GRIP model (P) (3), it is very challenging to solve it. Here, we propose to apply Boolean relaxation to P  (3), where we relax $u_{i}$ from {0,1} to [0, 1]. We use $P_{BR}$ to represent the relaxed problem of P  (3)


(4) $$\begin{array}{c} P_{BR}:\;\;\underset{u_{i}}{\min}:G(u):=y^{T}{\left(\frac{1}{\rho }XD(u)X^{T}+I\right)}^{-1}y+\lambda u^{T}Su\\ \;\;\;\;\;\;\;\;\;\;\;\;s.t.\sum_{i}u_{i}\leq k,\;u_{i}\in [0,1] \end{array}$$

In general, a relaxed model differs from the original model because certain constraints in the original problem are either loosened or removed. Therefore, the solution of the relaxed model might be very different from the original problem. However, the Boolean relaxation $P_{BR}$ has an attractive feature that is if the $\hat{u}\in {\left\{0,1\right\}}^{n}$ is the optimal solution of $P_{BR}$, then it must be the case that $\hat{u}=u^{*}$, where $u^{*}$ is the optimal solution of the original problem P. In another word, $P_{BR}=P$. In the following, we provide Theorem 1 to give the sufficient and necessary conditions for the Boolean relaxation $P_{BR}$ is tight, i.e. $P_{BR}=P$. The proof can be found in the Supplementary Material.

Theorem 1. *The Boolean relaxation is exact—that is*, $P_{BR}=P$  *– if and only if there exists a scalar* $\xi \in R^{+}$  *such that*
 

(5a) $${\left(x_{i}^{T}My\right)}^{2}+2\lambda \sum_{j}S_{ij}u_{j}^{*}>\xi \;\;\mathrm{for}\;\mathrm{all}\;i\in \Theta$$

 

(5b) $${\left(x_{i}^{T}My\right)}^{2}+2\lambda \sum_{j}S_{ij}u_{j}^{*}\leq \xi \;\;\mathrm{for}\;\mathrm{all}\;i\notin \Theta$$

*where* $M={\left(\frac{1}{\rho }XD(u)X^{T}+I\right)}^{-1}$, $u_{j}^{*}$  *is in the unique optimal solution* $u^{*}$  *of*  (3)  *and* Θ  *denotes the support of* $u^{*}$.

### 2.5 Solving the Boolean relaxation $P_{BR}$ of the GRIP model

It is easy to verify that the relaxed problem $P_{BR}$ is a convex optimization problem. The first term of the objective function in $P_{BR}$ is convex, proved by previous work (Pilanci *et al.* 2015), the second term $u^{T}Su$ is convex because *S* is positive definite (Cao *et al.* 2013), and the constraint set is convex. Therefore, $P_{BR}$ is convex. To efficiently solve $P_{BR}$, we propose to use the projected quasi-Newton method (Schmidt *et al.* 2009).

Two major computations are involved in the projected quasi-Newton method. The first is the computation of the derivative of the objective function G(u) of $P_{BR}$, which is given by


(6) $$\frac{dG}{du}=-\frac{1}{\rho }(X^{T}My)⊙(X^{T}My)+2\lambda Su$$

where $M={\left(\frac{1}{\rho }XD(u)X^{T}+I\right)}^{-1}$ and ⊙ represents the Hadamard product. The other major computation is the projection onto the constraint set $\left\{u|\sum_{i}u_{i}\leq k,\;u_{i}\in [0,1]\right\}$. Fortunately, in our previous work (Ang *et al.* 2021), we have developed an O(m) algorithm to efficiently compute such projection.

The Boolean relaxation $P_{BR}$  (4) is scalable. As mentioned above, the two major computations are the inverse of a m×m matrix $M={\left(\frac{1}{\rho }XD(u)X^{T}+I\right)}^{-1}$, where *m* is the number of cells, and the projection on the constraint set $\left\{u|\sum_{i}u_{i}\leq k,\;u_{i}\in [0,1]\right\}$. For the matrix inversion, if we have a large number *m* of cells, we can compute *M* by $M=I-\frac{1}{\rho }D(u)X{\left(I+D(u)X^{T}D(u)X\right)}^{-1}D(u)X^{T}$, which only involves the computation of the inversion of a n×n matrix ${\left(I+D(u)X^{T}D(u)X\right)}^{-1}$, where *n* is the number of TF candidates, and it is typical <1000. Therefore, the computation of *M* is very cheap. For the projection, in our previous work (Ang *et al.* 2021), we have developed an O(m) algorithm, which is scalable even if *m* (the number of cells) becomes large.

### 2.6 Randomized rounding of $P_{BR}$

The relaxed $P_{BR}$ is tight when Theorem 1 holds. However, there exist situations in which the solution of $P_{BR}$ is not Boolean but fractional. Here, we describe how to use fractional solution $\hat{u}\in {\left[0,1\right]}^{n}$ to produce a feasible Boolean solution $\tilde{u}\in {\left\{0,1\right\}}^{n}$.

We apply a simple form of randomized rounding to our relaxation $P_{BR}$. Given the fractional solution $\hat{u}\in {\left[0,1\right]}^{n}$, we generate a feasible Boolean solution $\tilde{u}\in {\left\{0,1\right\}}^{n}$ following the Bernoulli distribution


(7) $$P[{\tilde{u}}_{i}=1]={\hat{u}}_{i}\;\text{and}\;P[{\tilde{u}}_{i}=0]=1-{\hat{u}}_{i}$$

Through the rounding method proposed in (7), a random Boolean vector $\tilde{u}$ is generated. The random Boolean vector $\tilde{u}$ matches the fractional solution in expectation $E[\tilde{u}]=\hat{u}$ and moreover in expectation the number of selected ${\tilde{u}}_{i}$ ($\sum_{i=1}^{n}{\tilde{u}}_{i}$) is smaller than *k*, $E\left[\sum_{i=1}^{n}{\tilde{u}}_{i}\right]=\sum_{i=1}^{n}P[{\tilde{u}}_{i}=1]=\sum_{i=1}^{n}{\hat{u}}_{i}\leq k$.

Using the randomized rounding technique, we can generate several random Boolean vectors, and we choose the one, which gives the minimum objective function value $G(\tilde{u})$ in (4), as our final solution.

## 3 Results

We benchmark the GRIP model with state-of-the-art methods for inferring cell-type-specific gene regulation using scMultiome-seq data and PCHiC data.

### 3.1 Experiment setup

#### 3.1.1 Competing methods

There are many state-of-the-art methods using paired gene expression and chromatin accessibility data. These methods include Scenic+ (Bravo González-Blas *et al.* 2023), Pando (Fleck *et al.* 2022), CellOracle (Kamimoto *et al.* 2023), DirectNet (Zhang *et al.* 2022), GRaNIE (Kamal *et al.* 2023), FigR (Kartha *et al.* 2022), and LINGER (Yuan and Duren 2024). Previous study (Bravo González-Blas *et al.* 2023) showed that Scenic+ (Bravo González-Blas *et al.* 2023) significantly outperforms GRaNIE (Kamal *et al.* 2023) and FigR (Kartha *et al.* 2022); therefore, we exclude them in our comparison. LINGER (Yuan and Duren 2024) uses atlas-scale external data to pre-train the model. For a fair comparison, we exclude LINGER (Yuan and Duren 2024) in our comparison as well as in the main text. However, we have reported the comparison between GRIP and LINGER in Supplementary Material.

Therefore, in all the experiments in this article, we benchmark our GRIP model with Scenic+ (Bravo González-Blas *et al.* 2023), Pando (Fleck *et al.* 2022), CellOracle (Kamimoto *et al.* 2023), and DirectNet (Zhang *et al.* 2022).

#### 3.1.2 Data

We use scMultiome-seq PBMC 3k data (10x Genomics 2021) in our experiments. Specifically, we extract the paired scRNA-seq and scATAC-seq data of CD4 T cells, CD8 T cells, and CD14+ monocytes from the scMultiome-seq PBMC 3k data, respectively. We apply all the competing methods on the extracted data to infer gene regulation for CD4 T cells, CD8 T cells, and CD14+ monocytes.

For the scRNA-seq data, we use regular pipeline (Wolf *et al.* 2018) to process the data following the legacy workflow provided by Scanpy (https://scanpy.readthedocs.io/en/stable/tutorials/basics/clustering-2017.html). For the scATAC-seq data, any peak within 500 bp upstream of a TG’s transcription start site is defined as the promoter of the gene, while other open chromatin regions outside of the promoter region but within 250 kb on both sides are defined as distal candidate functional regions.

To run the GRIP model, we also need a PPI network as input. Therefore, we download the PPIs from BioGRID (Oughtred *et al.* 2019) and extract only the physical interactions to construct the PPI network for the GRIP model.

#### 3.1.3 PCHiC data

We use PCHiC data (Javierre *et al.* 2016) of naive CD4 T cells, naive CD8 T cells, and monocytes to evaluate all the competing methods. PCHiC stands for Promoter Capture Hi-C, which focuses on capturing the physical interactions between distant genomic regions and gene promoters. For the PCHiC data of naive CD4 T cells, we extract 2405 links between 411 TGs’ promoters and their distant chromatin open regions. For the PCHiC data of naive CD8 T cells, we extract 2540 links between 422 TGs’ promoters and their distant chromatin open regions. For the PCHiC data of monocytes, we extract 2131 links between 323 TGs’ promoters and their distant chromatin open regions.

#### 3.1.4 Evaluation metrics

It is very challenging to evaluate the gene regulation inference because we do not have the ground truth. Therefore, we propose three metrics to evaluate the performance of the competing methods. The three metrics are out-of-sample mean square error (OOS MSE), average TF distance, and agreement between PCHiC data and the inferred gene regulations. We should not evaluate the method based on only one metric but consider all three. For a good method, we expect, for most of the TGs, the inferred TFs predicted by the good method should achieve smaller OOS MSE, smaller average TF distance, and better agreement between PCHiC data and the inferred gene regulations.

*OOS MSE*: The first metric we use is the out-of-sample mean square error (OOS MSE). OOS MSE measures the predictive power of the TFs for a TG. As described in a previous study (Yuan and Duren 2024), the gene expression of a TG can be predicted by the gene expression of its underlying TFs. To measure OOS MSE in our experiment, we first split the cell-type-specific scMultiome-seq data into the training data and testing data. The training data contains 90% of the scMultiome-seq data, and the testing data contains the rest of the 10%. For all the competing methods, we first use the training data to infer the TFs for TGs. Next, we use the testing data to measure the predictive power of the inferred TFs for TGs. Using a similar idea in a previous study (Yuan and Duren 2024), we build a three-layer neural network to model the gene expression of a TG using the gene expression of its inferred TFs. To avoid over-fitting, we set the number of neurons for three layers to be the number of inferred TFs, 20, and 1, respectively. We use the training data to train the neural network and compute the OOS MSE on the testing data. The smaller the OOS MSE, the better predictive power the inferred TFs have. For each TG, we can compute the OOS MSE.

*Remark for OOS MSE*: OOS MSE measures the out-of-sample performance. The smaller the OOS MSE of a method, the better the method predicts the expression of the TGs. However, OOS MSE would not decrease when one method includes more TFs in the prediction.

*TF distance*: The second metric we use is the average TFs distance between the inferred TFs of TGs. As we proved in Section 2.2, TFs regulating the same TGs should stay close in a PPI network. For a TG, we compute the pairwise DSD distance (Cao *et al.* 2013) between the inferred TFs and then compute the average. For each TG, we can compute the average TFs distance. The goal of using this metric is to check whether our model can identify TFs that are close to each other compared to other methods.

*F1 score*: The third metric we use is the *F*1 score to measure the agreement between the inferred gene regulation and the PCHiC data. We know *F*1$=2\frac{\text{precision}\times \text{recall}}{\text{precision}+\text{recall}}$. Therefore, we need to compute precision and recall to compute the *F*1 score. For a given TG, we know the PCHiC links, which represent the open regions with which the promoter of the TG interacts. We put these open regions in set Λ. To compute the *F*1 score for the TG, we can first retrieve a set Ω containing all the chromatin open regions that the inferred TFs could bind based on the motif-matching information of the inferred TFs. We can find a subset Δ of Ω, which contains the chromatin open regions that overlap with the PCHiC links for the same TG. The precision can then be computed by $\frac{\left|\Delta \right|}{\left|\Omega \right|}$. Furthermore, we can compute the recall by $\frac{\left|\Delta \right|}{\left|\Lambda \right|}$. For each TG, we can compute an *F*1 score.

Remark for *F*1 score: For a TG, the number of TFs inferred by the competing methods would be different. To avoid the influence brought by the different number of TFs, we use F1_top*k* to fairly evaluate the competing methods. F1_top*k* is the *F*1 score when considering the top *k* predicted TFs. F1_top*k* ensures to compare the competing methods in terms of the top *k* inferred TFs.

#### 3.1.5 Hyper-parameter tuning

The GRIP model (4) has three hyper-parameters, which are λ, ρ, and *k*. For all the experiments in the article, we use cross-validation to select the optimal hyper-parameters to avoid the influence of the parameter selection. All other competing methods provide tutorials using PBMC 3k scMultiome-seq data. Therefore, we just use the default hyper-parameters provided by their tutorials (Fleck *et al.* 2022, Zhang *et al.* 2022, Bravo González-Blas *et al.* 2023, Kamimoto *et al.* 2023).

### 3.2 GRIP achieves smaller OOS MSE

In this section, we apply all the competing methods to infer gene regulation for CD4 T cells, CD8 T cells, and CD14 monocytes using PBMC 3k scMultiome-seq data. Furthermore, we evaluate the competing methods in terms of the predictive power of the inferred TFs using OOS MSE (the first metric described in Section 3.1).

**Figure 4.**
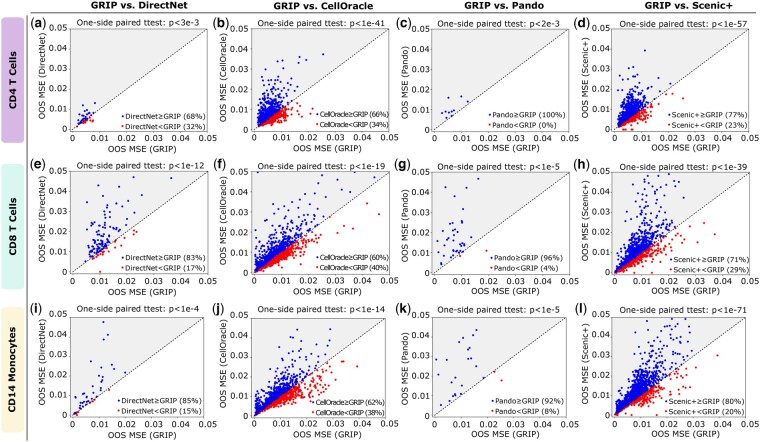
The comparison of OOS MSE for all competing methods on CD4 T cells, CD 8 T cells, and CD14 monocytes. Each point in each plot represents a TG. The coordinates of the point/TG are the OOS MSE values for the TG computed by the compared methods. On top of each plot, we provide the *P*-value from the one-side paired *t*-test to show whether the OOS MES achieved by the GRIP model is significantly smaller than the competing method. Only TGs that are common in both competing methods are shown in the plots. Gray-colored regions indicate GRIP outperforms the competing methods. (a–d) Pairwise comparison between the GRIP model and others for CD4 T cells. (e–h) Pairwise comparison between the GRIP model and others for CD8 T cells. (i–l) Pairwise comparison between the GRIP model and others for CD14 monocytes.

Figure 4a–d illustrates the pairwise comparison between GRIP and other competing methods for CD4 T cells. Each point in the plots represents a TG. Notably, the numbers of TGs (points) in Fig. 4a–d are different. The reason is that although working on the same data, different methods make inferences for different sets of TGs in their results. For a fair comparison, we have plotted only those TGs that are common between the two methods being compared in each case. As shown in Fig. 4a–d, the OOS MSE achieved by GRIP is significantly smaller than the competing methods (*P*-values can be found in Fig. 4a–d). Furthermore, we find that the TFs inferred by our GRIP model make 68% of TGs in Fig. 4a, 66% of TGs in Fig. 4b, 100% of TGs in Fig. 4c, and 77% of TGs in Fig. 4d achieve smaller OOS MSE.

Figure 4e–h illustrates the pairwise comparison between GRIP and the competing methods for CD8 T cells. As shown in Fig. 4e–h, the OOS MSE achieved by GRIP is significantly smaller than the competing methods (*P*-values can be found in Fig. 4e–h). Furthermore, we find that the TFs inferred by our GRIP model make 83% of TGs in Fig. 4e, 60% of TGs in Fig. 4f, 96% of TGs in Fig. 4g, and 71% of TGs in Fig. 4h achieve smaller OOS MSE.

Figure 4i–l illustrates the pairwise comparison between GRIP and the competing methods for CD14 monocytes. As shown in Fig. 4i–l, the OOS MSE achieved by GRIP is significantly smaller than the competing methods (*P*-values can be found in Fig. 4i–l). Furthermore, we find that the TFs inferred by our GRIP model make 85% of TGs in Fig. 4i, 62% of TGs in Fig. 4j, 92% of TGs in Fig. 4k, and 80% of TGs in Fig. 4l achieve smaller OOS MSE.

We have also compared GRIP with LINGER in terms of OOS MSE and reported the results in Supplementary Material. LINGER outperforms GRIP on CD4 cells. But, GRIP outperforms LINGER on CD8 and CD 14 cells.

In sum, the comparison results shown in this section demonstrate that the TFs inferred by the GRIP model have more predictive power than other competing methods.

### 3.3 TFs inferred by GRIP are closer in the PPI network

In this section, we compare the distance between TFs inferred by the competing methods. We use the average TFs distance as the metric (described in Section 3.1) to evaluate the performance of the competing methods. The detailed comparison is shown in Supplementary Material. In sum, the comparison results demonstrate that the TFs inferred by the GRIP model are closer in the PPI network, which is expected because GRIP directly considers the TFs distance in the model.

### 3.4 GRIP achieves better *F*1 score

In this section, we compare the F1_top3 scores (the third metric described in Section 3.1) of the competing methods using PCHiC data (Javierre *et al.* 2016). F1_top3 is the *F*1 score when considering the top three inferred TFs. Using F1_top3 would remove the influence incurred by the inconsistency of the number of TFs inferred for a TG by different methods. We also provide F1_top5 score comparisons in the Supplementary Material.

**Figure 5.**
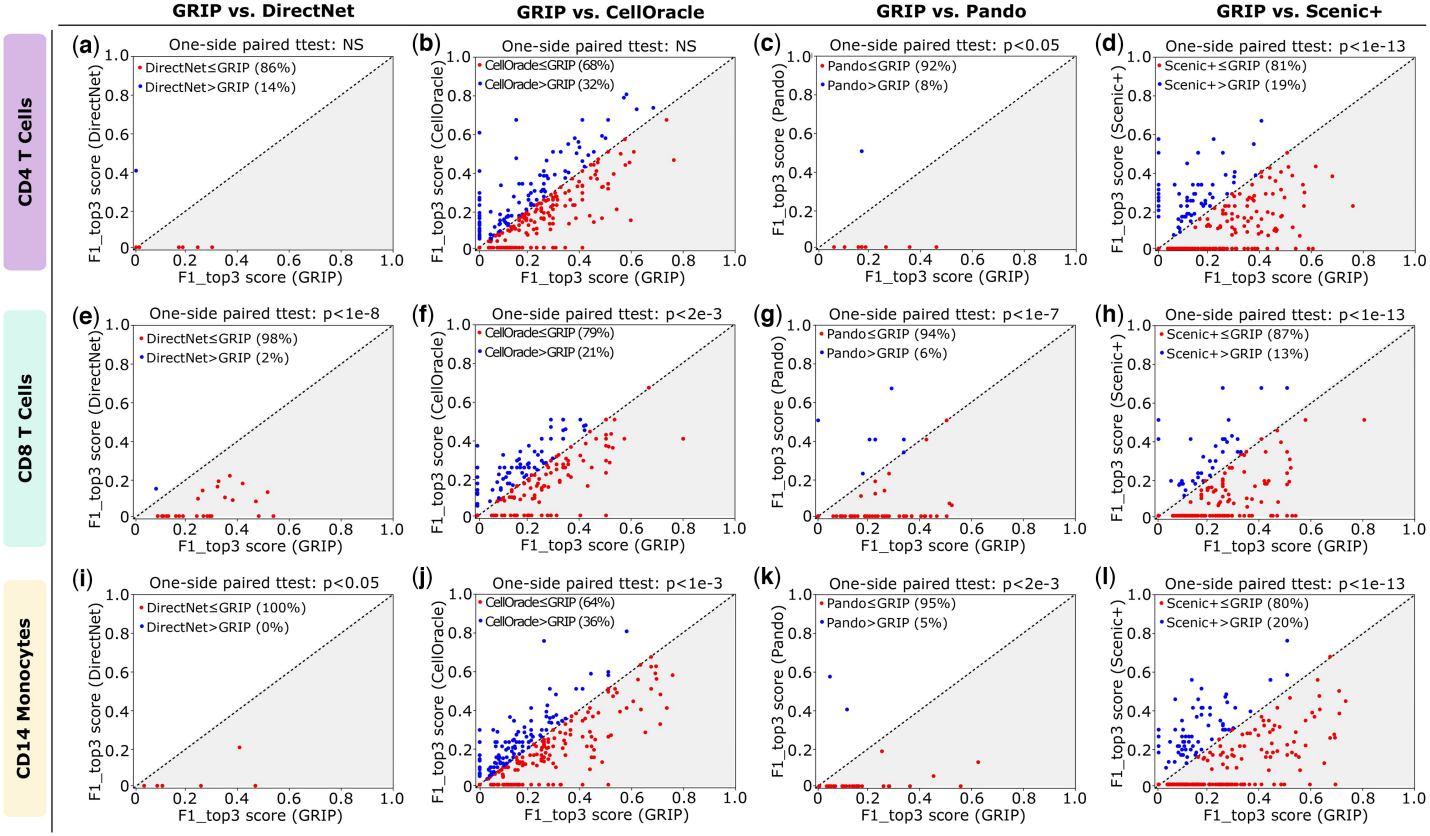
The comparison of F1_top3 score (the third metric described in Section 3.1) for all competing methods on CD4 T cells, CD 8 T cells, and CD14 monocytes. Each point in each plot represents a TG. The coordinates of the point/TG are the F1_top3 scores for the TG computed by the compared methods. On top of each plot, we provide the *P*-value from the one-side paired *t*-test to show whether the F1_top3 scores achieved by the GRIP model are significantly larger than the competing method. Only TGs that are common in both competing methods are shown in the plots. Gray-colored regions indicate GRIP outperforms the competing methods. (a–d) Pairwise comparison between the GRIP model and others for CD4 T cells. (e–h) Pairwise comparison between the GRIP model and others for CD8 T cells. (i–l) Pairwise comparison between the GRIP model and others for CD14 monocytes. In the plots, you might see for some TGs, the competing methods achieve *F*1 = 0, meaning all their prediction are false positives.

Figure 5a–d illustrates the pairwise comparison between GRIP and competing methods for CD4 T cells. As shown, GRIP achieves significantly larger F1_top3 scores compared to Pando and Scenic+. Compared with DirectNet and CellOracle, although the *P*-value is not significant, for the majority of the TGs (86% TGs compared with DirectNet and 68% TGs compared with CellOracle), GRIP achieves larger F1_top3 scores.

Figure 5e–h illustrates the pairwise comparison between GRIP and competing methods for CD8 T cells. As shown, GRIP achieves significantly larger F1_top3 scores compared to all the competing methods. Furthermore, GRIP achieves larger F1_top3 scores for 98% TGs compared with DirectNet, 79% TGs compared with CellOracle, 94% TGs compared with Pando, and 87% TGs compared with Scenic+.

Figure 5i–l illustrates the pairwise comparison between GRIP and competing methods for CD14 monocytes. As shown, GRIP achieves significantly larger F1_top3 scores compared to all the competing methods. Furthermore, GRIP achieves larger F1_top3 scores for 100% TGs compared with DirectNet, 64% TGs compared with CellOracle, 95% TGs compared with Pando, and 80% TGs compared with Scenic+.

We have also compared GRIP with LINGER in terms of F1_top3 score and reported the results in the Supplementary Material. We found GRIP outperforms LINGER on CD4, CD8, and CD14 cells.

In sum, the F1_top3 score comparison results demonstrate that the gene regulation inferred by GRIP is in better agreement with the PCHiC data compared with the competing methods. Based on the comparison in terms of the F1_top5 score (details in the Supplementary Material), we have a similar observation.

### 3.5 Benchmark on CD8 cells using PBMC 10k data

We have benchmarked all competing methods using a larger dataset (PBMC 10k data). Specifically, we have compared GRIP with all competing methods on gene regulation inference for CD8 cells using PBMC 10k data (10k cells). We illustrated the comparison results in Supplementary Material. We find that GRIP outperforms all competing methods in terms of the three metrics.

### 3.6 Ablation study (GRIP versus GRIP(λ=0))

**Figure 6.**
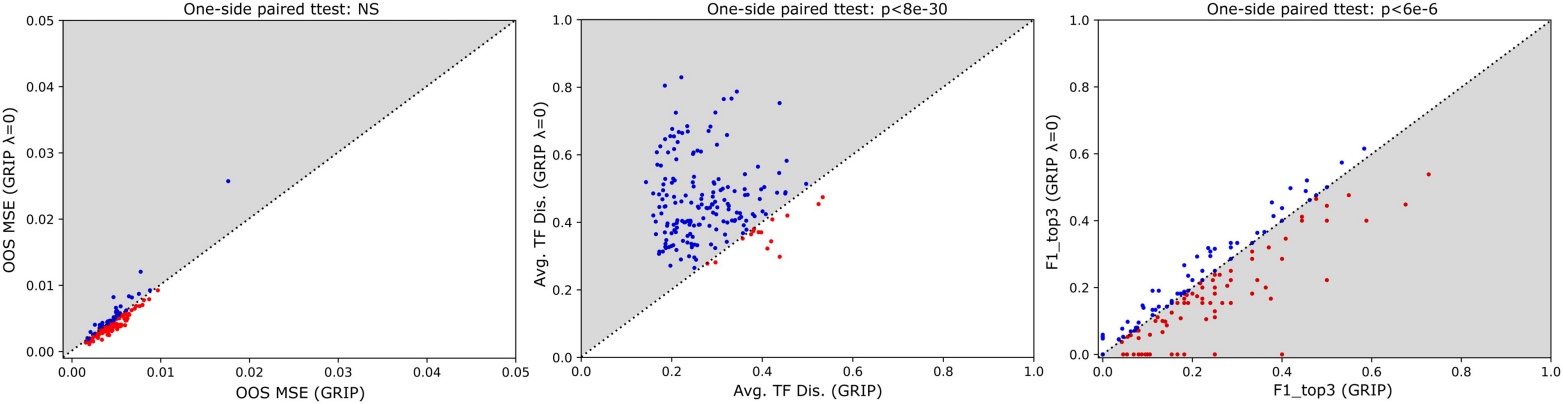
The comparison between GRIP and GRIP (λ=0) on CD4 T cells in terms of the three metrics. Each dot represents a TG. The coordinates of each dot are the corresponding metric values for GRIP and GRIP (λ=0), respectively. Gray-colored regions indicate GRIP outperforms GRIP (λ=0).

In this section, we check whether considering TF proximity helps with the gene regulation inference in GRIP. We compared the GRIP model’s results reported above on CD4 T cells with the GRIP model’s results when we set λ=0 (in other words, we do not consider TF proximity in the GRIP model) in terms of the three metrics. We use cross-validation to select the optimal λ for our GRIP model (as described in Section 3.1). We found that the optimal λ for 989 TGs out of 1753 TGs is not 0, which implies that TF proximity helps with the gene regulation inference for these 989 TGs. We compared the GRIP model with the GRIP model (λ=0) for these 989 TGs in terms of the three metrics described in Section 3.1. As shown in Fig. 6, GRIP and GRIP (λ=0) are comparable in terms of OOS MSE. But GRIP outperforms GRIP (λ=0) in terms of average TF distance and F1_top3 score, which indicates that TF proximity indeed helps with the gene regulation inference.

We have conducted an additional ablation study to compare the GRIP model using the BioGRID PPI network and the GRIP model using a random PPI network generated from the BioGRID PPI network using degree-preserving randomization (Maslov and Sneppen 2002). We report the results in Supplementary Material. We find that the GRIP model using the BioGRID PPI network outperforms the GRIP model using a random PPI network in terms of the three metrics. Furthermore, we observe that using a random PPI network performs worse than not using any PPI information.

## 4 Discussion

Gene regulation inference has been one of the key challenges in computational biology. Existing methods do not consider the physical proximity of the TFs when inferring gene regulation. Here, we utilize a PPI network and propose a novel Boolean convex program to identify TFs that not only can explain the gene expression of TGs but also stay close in the PPI network. Furthermore, we prove that the Boolean relaxation of the original Boolean convex program is tight (Theorem 1 provides the necessary and sufficient conditions) and can be efficiently solved by the projected quasi-Newton method. We believe that the novel Boolean convex program presented in this study provides not only an important step toward reconstructing better gene regulatory networks but also has the potential to become a paradigm for addressing other problems in computational biology.

There are also some limitations in our GRIP model. GRIP relies on the hypothesis/assumption that the physical proximity of TFs during transcription initiation is well characterized by PPI networks. However, this might not be the case if there are proteins that influence gene expression independently, e.g. by influencing chromatin accessibility. Also, PPI networks collect information on whether two proteins interact with each other in specific experimental conditions that might not resemble those of transcription initiation.

## Supplementary Material

btaf186_Supplementary_Data

## Data Availability

No new data were generated or analysed in support of this research.
