# Supplementary material for: Leveraging transcription factor physical proximity for enhancing gene regulation inference
Source: Bioinformatics. 2025 Jul 15;41(Suppl 1):i533–41. doi: 10.1093/bioinformatics/btaf186 (PMC12261443; doi:10.1093/bioinformatics/btaf186)
Supplement: btaf186_Supplementary_Data [file btaf186_supplementary_data.zip › Supplementary_Materials.pdf]

Supplementary Materials

A Proof Theorem 1

Theorem 1. *The Boolean relaxation is exact – that is, $\mathcal{P}_{BR} = \mathcal{P}$ – if and only if there exists a scalar $\xi \in \mathbb{R}^+$ such that*

$$\left(x_i^T My\right)^2 + 2\lambda \sum_j S_{ij} u_j^* > \xi \quad for\ all\ i \in \Theta \tag{1a}$$

$$\left(x_i^T My\right)^2 + 2\lambda \sum_j S_{ij} u_j^* \leq \xi \quad for\ all\ i \notin \Theta, \tag{1b}$$

*where $M = (\frac{1}{\rho} X D(\boldsymbol{u}) X^T + I)^{-1}$, u_j^* is in the unique optimal solution $\boldsymbol{u}^*$ of (**??**) and Θ denotes the support of $\boldsymbol{u}^*$.*

We use the following two Theorems to prove Theorem 1.

Theorem 2 ([1]). *Suppose $\bar{x}$ is a local minimizer of $f : \mathbb{R}^d \to \mathbb{R}$ on a closed convex set $\mathcal{X} \subseteq \mathbb{R}^d$. If f is differentiable at $\bar{x}$, it holds that*

$$-\nabla f(\bar{x}) \in \mathcal{N}_{\mathcal{X}}(\bar{x}). \tag{2}$$

Theorem 3 ([1]). *Let $A \in \mathbb{R}^{m \times n}$ and let $\beta \in \mathbb{R}^m$. Consider the polyhedron $Q(A, \beta) = \{x | Ax \leq \beta\}$. Suppose $x \in Q(A, \beta)$, then the normal cone at x is $N_{Q(A,\beta)}(x) = \{A^\top y | y \in \mathbb{R}^m$ such that $y \geq 0$ and $y^\top (\beta - Ax) = 0\}$.*

Proof Theorem 1. We apply the first-order convex optimality condition (Theorem 2) for constrained minimization to our model (**??**). To use (2). We first derive the left-hand side and get

$$\frac{\partial G(u)}{u_i} = -\frac{1}{\rho}(x_i^T My)^2, \tag{3}$$

where $M = (\frac{1}{\rho} X D(\boldsymbol{u}) X^T + I)^{-1}$. The right-hand side of (2) is the normal cone of the constraint set of our model (**??**). Because the constraint set of our model (**??**) are linear constraints, we can get the Theorem 3 to find the normal cone.

Combining the derivative of the objective of (**??**) and the normal cone of the constraints in (**??**), we obtain

$$\xi_{u_i \leq 1} - \xi_{u_i \geq 0} + \xi_k = \left(x_i^T My\right)^2 + 2\lambda \sum_j S_{ij} u_j, \tag{4}$$

where $\xi_{u_i \leq 1}$, $\xi_{u_i \geq 1}$, and ξ_k (dual variable for $\sum_i u_i \leq k$) are the dual variables for the constraints in (**??**).

Let $\boldsymbol{u}^*$ represent the unique optimal solution of (**??**). If $u_i^* = 1$ is in the unique optimal solution, then $\xi_{u_i \leq 1} > 0$ and $\xi_{u_i \leq 1} = 0$. Therefore, we have

$$\xi_k < \left(x_i^T My\right)^2 + 2\lambda \sum_j S_{ij} u_j^*, \tag{5}$$

Otherwise, if $u_i^* = 0$ is in the unique optimal solution, then $\xi_{u_i \leq 1} = 0$ and $\xi_{u_i \leq 1} > 0$. Therefore, we have

$$\xi_k \geq \left(x_i^T M y\right)^2 + 2\lambda \sum_j S_{ij} u_j^*, \tag{6}$$

Replacing ξ_k with ξ proves the Theorem. □

B Ablation study

We have conducted an additional ablation study to compare the GRIP model using the BioGRID PPI network and the GRIP model using a random PPI network generated from the BioGRID PPI network using degree-preserving randomization [2]. As shown in Fig. S1, the GRIP model using the BioGRID PPI network outperforms the GRIP model using a random PPI network in terms of the three metrics. For comparison of TF distance (the middle plot in Fig. S1), we found that for all TGs, the distance of the inferred TFs identified by the GRIP model using the BioGRID PPI network is smaller than using a random PPI network. The reason is that when computing the TF distance, we used the BioGRID PPI network. After comparing with Fig.S1 and Fig. 6 in the main text, we found that using a random PPI network performs worse than not using any PPI information.

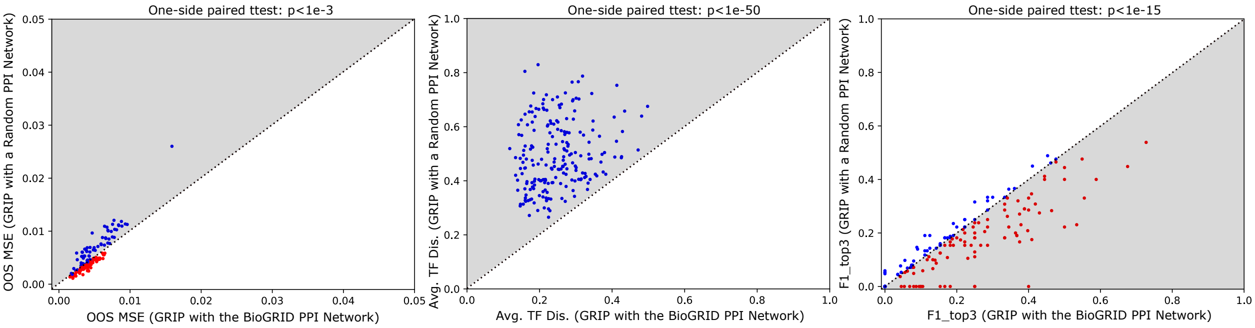

Fig. S1: The ablation study compares the GRIP model using the BioGRID PPI network and the GRIP model using a random PPI network generated from the BioGRID PPI network using degree-preserving randomization.

C F1_top5 comparison

In the main manuscript, we show the F1_top3 comparison between the competing methods, where the F1 score is computed based on the top 3 predicted TFs for each TG. Here, we illustrate similar comparison results in terms of F1_top5 as shown in Fig. S2, where the F1 score is computed based on the top 5 predictions. As shown in Fig. S2, we have a similar observation to what we have in the main manuscript: our GRIP model outperforms other competing methods in terms of F1_top5.

D TFs inferred by GRIP are closer in the PPI network

In this section, we compare the distance between TFs inferred by the competing methods. We use the average TFs distance as the metric (described in section ??) to evaluate the performance of the competing methods.

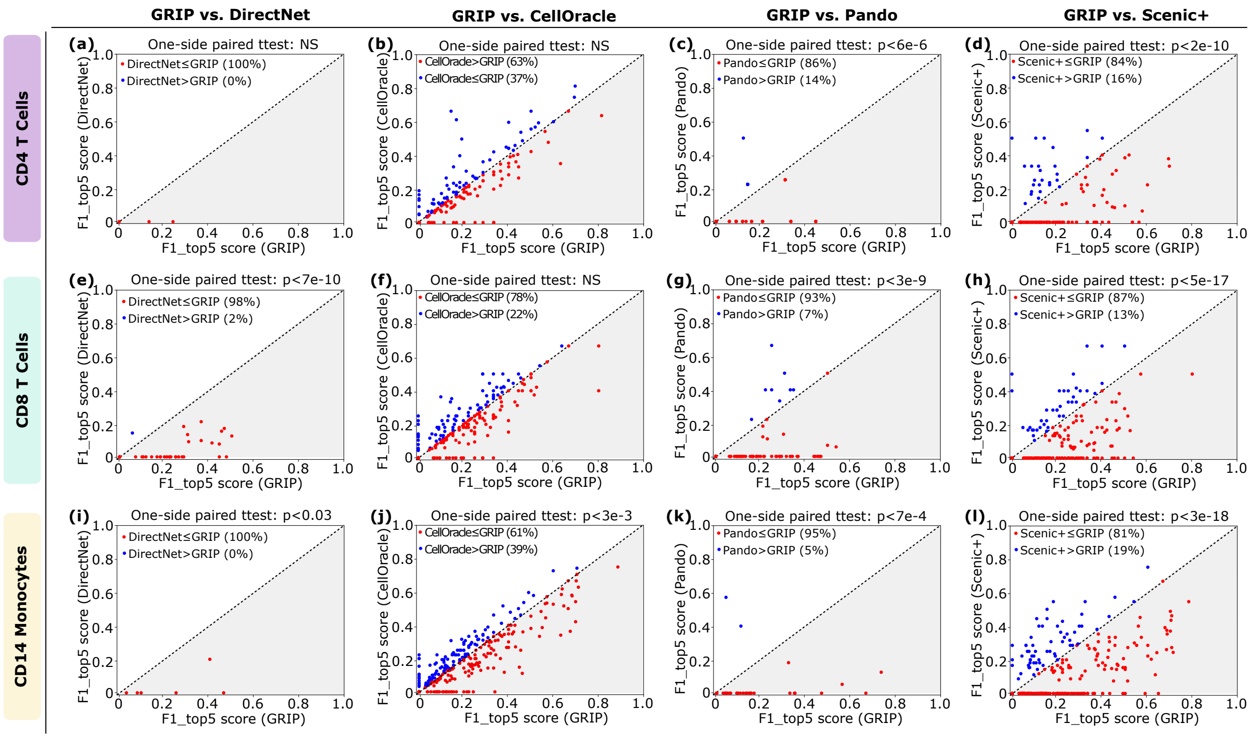

Fig. S2: The comparison of F1_top5 score (the third metric described in section **??**) for all competing methods on CD4 T cells, CD 8 T cells, and CD14 monocytes. Each point in each plot represents a TG. The coordinates of the point/TG are the F1_top3 scores for the TG computed by the compared methods. Gray-colored regions indicate GRIP outperforms the competing methods. On top of each plot, we provide the p-value from the one-side paired t-test to show whether the F1_top5 scores achieved by the GRIP model are significantly larger than the competing method. Only TGs that are common in both competing methods are shown in the plots. (a-d) Pairwise comparison between the GRIP model and others for CD4 T cells. (e-h) Pairwise comparison between the GRIP model and others for CD8 T cells. (i-l) Pairwise comparison between the GRIP model and others for CD14 monocytes. In the plots, you might see for some TGs, the competing methods achieve F1=0, meaning all their prediction are false positives.

Fig. S3(a-d) illustrate the pairwise comparison between GRIP and competing methods for CD4 T cells in terms of average TFs distance. As shown in Fig. S3(a-d), the average TFs distance achieved by GRIP is significantly smaller than the competing methods (p-values can be found in Fig. S3(a-d)). Furthermore, we find that 95% of TGs in Fig. S3(a), 89% of TGs in Fig. S3(b), 80% of TGs in Fig. S3(c), and 80% of TGs in Fig. S3(d), whose regulating TFs identified by GRIP have smaller distance in a PPI network.

Fig. S3(e-h) illustrate the pairwise comparison between GRIP and competing methods for CD8 T cells. As shown in Fig. S3(e-h), the average TFs distance achieved by GRIP is significantly smaller than the competing methods (p-values can be found in Fig. S3(e-h)). Furthermore, we find that 88% of TGs in Fig. S3(e), 93% of TGs in Fig. S3(f), 98% of TGs in Fig. S3(g), and 91% of TGs in Fig. S3(h), whose regulating TFs identified by GRIP have smaller distance in a PPI network.

Fig. S3(i-l) illustrate the pairwise comparison between GRIP and other competing methods for CD14 monocytes. As shown in Fig. S3(i-l), the average TFs distance achieved by GRIP is significantly smaller than the competing methods (p-values can be found in Fig. S3(i-l)). Furthermore, we find that 88% of TGs in Fig. S3(i), 94% of TGs in Fig. S3(j), 92% of TGs in Fig. S3(k), and 92% of TGs in Fig. S3(l), whose regulating TFs identified by GRIP have smaller distance in a PPI network.

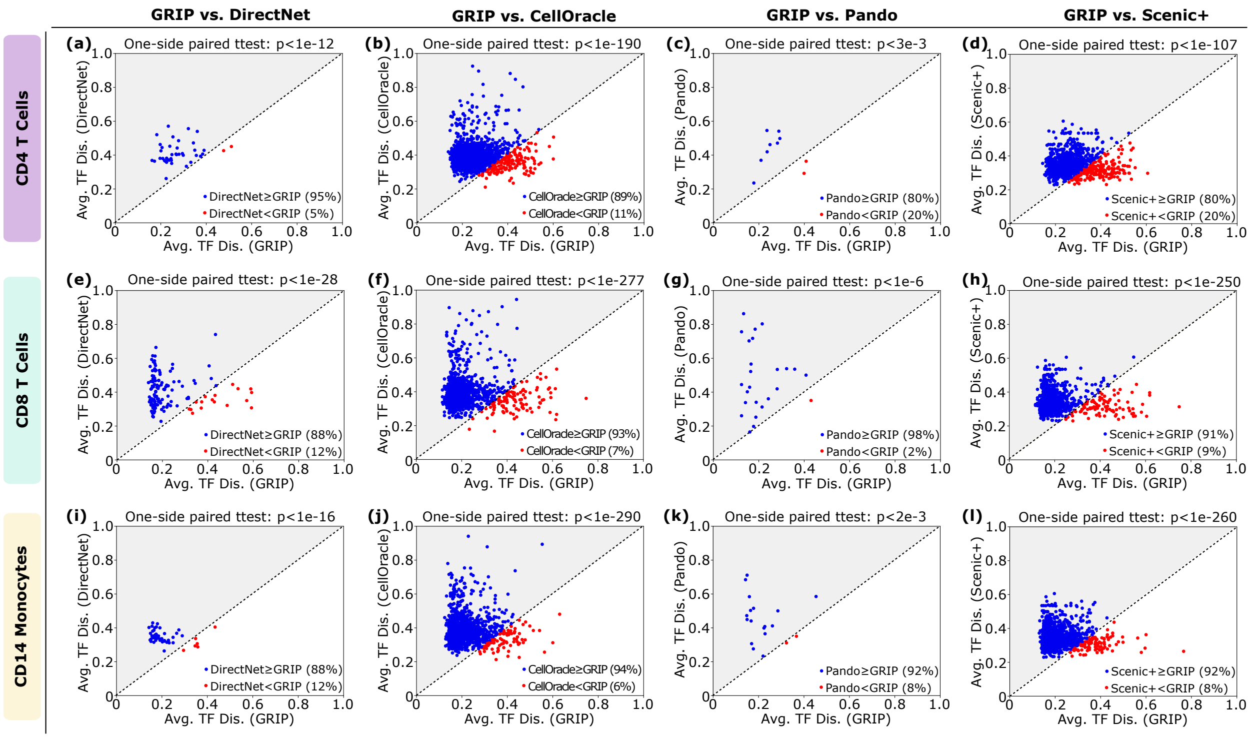

Fig. S3: The comparison of avg. TFs distance (the second metric described in section ??) for all competing methods on CD4 T cells, CD8 T cells, and CD14 monocytes. Each point in each plot represents a TG. The coordinates of the point/TG are the avg. TFs distance values for the TG computed by the compared methods. On top of each plot, we provide the p-value from the one-side paired t-test to show whether the avg. TFs distance achieved by the GRIP model is significantly smaller than the competing method. Only TGs that are common in both competing methods are shown in the plots. Gray-colored regions indicate GRIP outperforms the competing methods. (a-d) Pairwise comparison between the GRIP model and others for CD4 T cells. (e-h) Pairwise comparison between the GRIP model and others for CD8 T cells. (i-l) Pairwise comparison between the GRIP model and others for CD14 monocytes.

In sum, the comparison results demonstrate that the TFs inferred by the GRIP model are closer in the PPI network, which is expected because GRIP directly considers the TFs distance in the model.

E Benchmarking with LINGER [3]

Furthermore, we have compared our GRIP with LINGER [3] on gene regulation inference for CD4, CD8, and CD14 cells using PBMC 3k data in terms of the three metrics proposed in the manuscript (as shown in Fig. S4). We find that for CD8 and CD14 cells, GRIP outperforms LINGER in terms of the three metrics. For the comparison on CD4 cells, LINGER outperforms GRIP in terms of OOS MSE (Fig. S4 (a)). But GRIP outperforms LINGER in terms of TFs distance and F1_top3 scores (Fig. S4 (b-c)).

F Benchmarking on CD8 using PBMC 10k data [4]

we have compared with all the competing method using a larger dataset. Specifically, we have compared GRIP with all the competing methods including Scenic+, Pando, CellOracle, DirectNet, and LINGER on gene regulation inference for CD8 cells using PBMC 10k data [4]

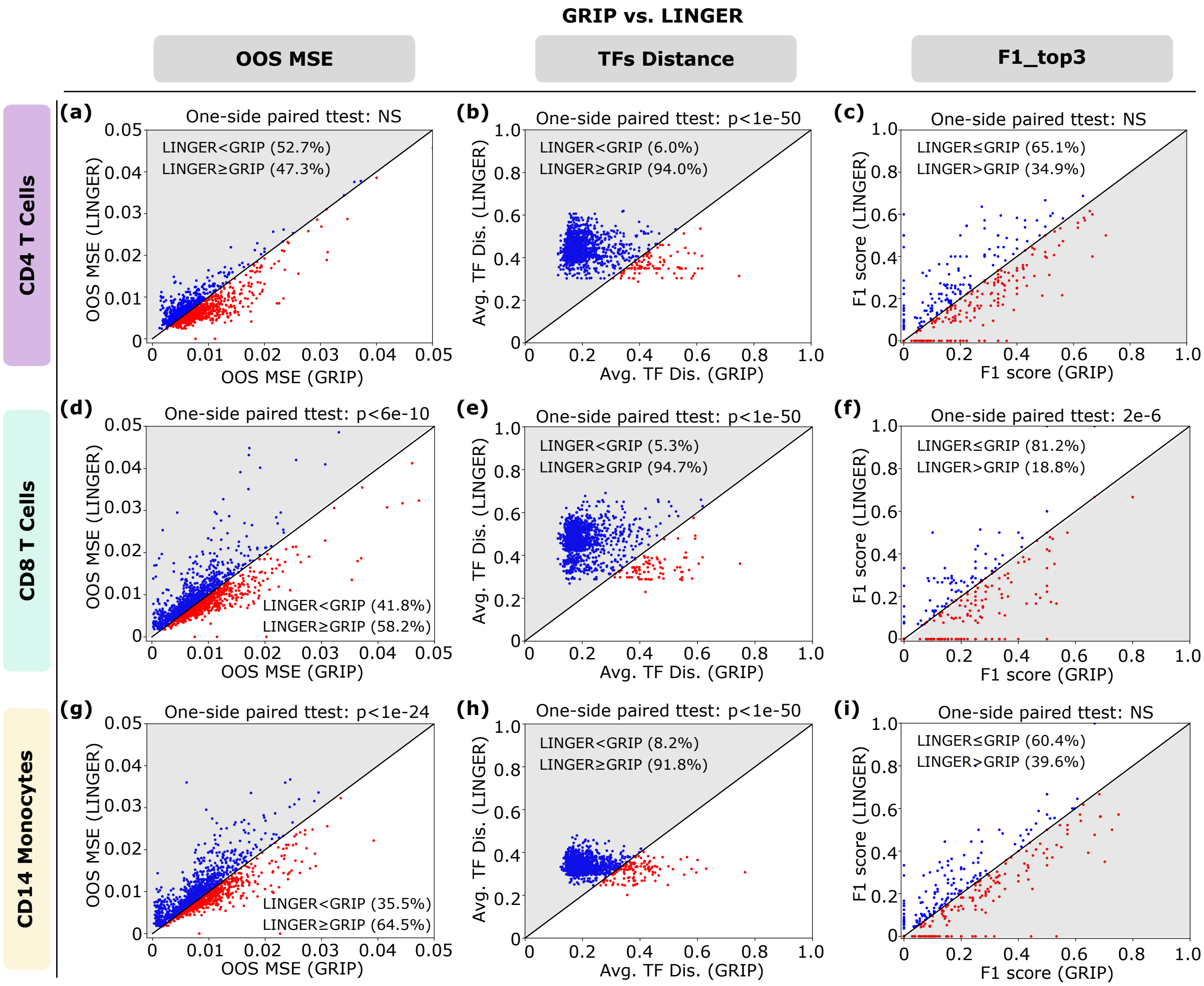

Fig. S4: The comparison between GRIP and LINGER on inferring gene regulation for CD4, CD8, and CD14 cells using PBMC 3k data. Each point in each plot represents a TG. On top of each plot, we provide the p-value from the one-side paired t-test to show whether the scores achieved by the GRIP are significantly larger than the competing method. Only TGs that are common in both competing methods are shown in the plots. Gray-colored regions indicate GRIP outperforms the competing methods. For all the F1_top3 comparisons, GRIP only compares with LINGER for TGs whose F1_top3 is larger than 0. (a-c) The comparison between GRIP and LINGER on CD4 cells in terms of the three metrics. (d-f) The comparison between GRIP and LINGER on CD8 cells in terms of the three metrics. (g-i) The comparison between GRIP and LINGER on CD14 cells in terms of the three metrics.

(1̃0k cells). We illustrate the comparison results in Fig. S5. As shown, GRIP outperforms all the competing methods in terms of the three metrics.

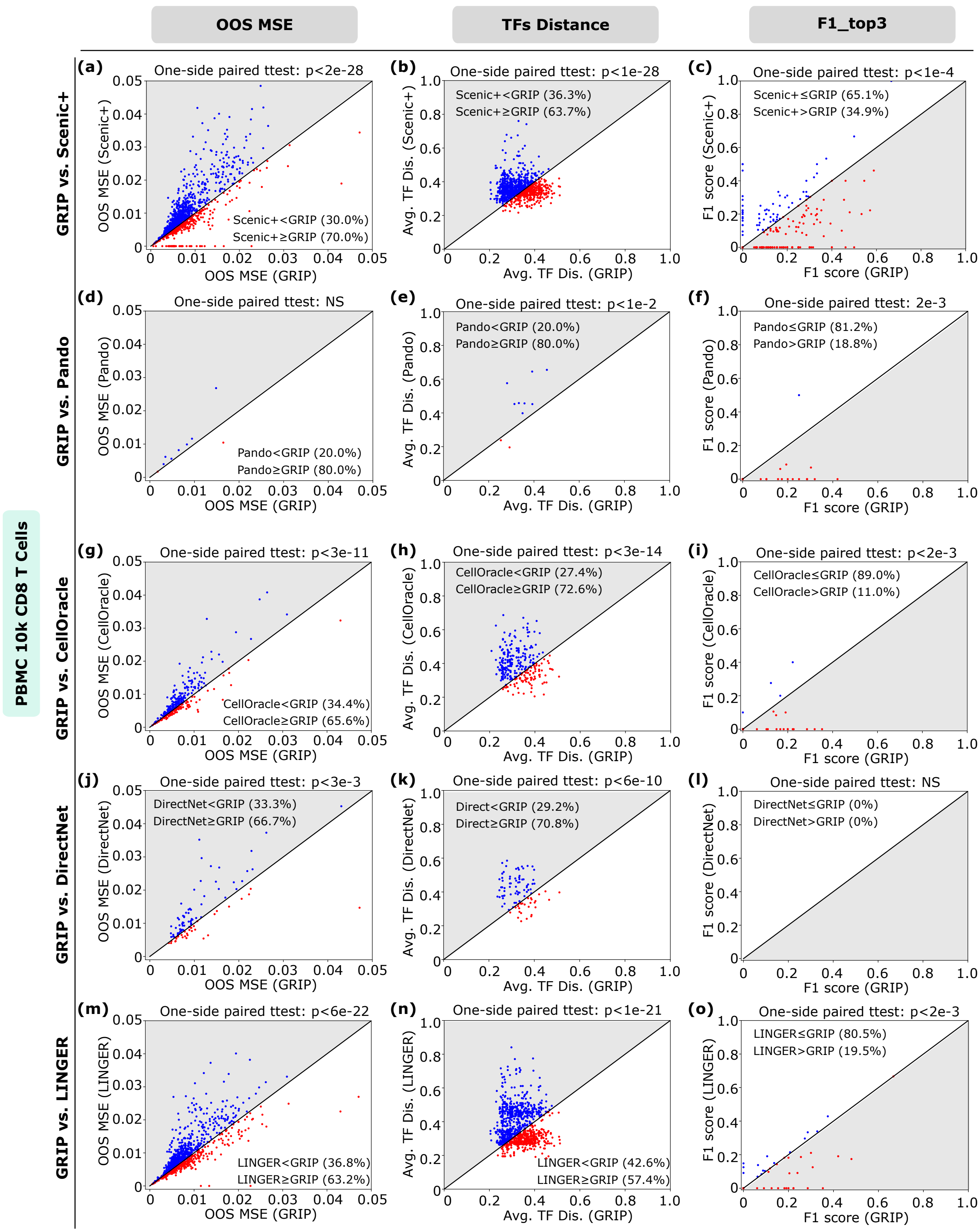

Fig. S5: The comparison between GRIP and LINGER on inferring gene regulation for CD8 cells using PBMC 10k data in terms of the three metrics. (a-c) The comparison between GRIP and Scenic+ in terms of the three metrics. (d-f) The comparison between GRIP and Pando in terms of the three metrics. (g-i) The comparison between GRIP and CellOracle in terms of the three metrics. (j-l) The comparison between GRIP and DirectNet in terms of the three metrics. The F1_top3 scores for the regulation of the target genes generated by DirectNet are all zeros. So, there is no point in (l). (m-o) The comparison between GRIP and LINGER in terms of the three metrics.
